# Somatic substitution signature as an innovative tool in lung cancer diagnosis

**DOI:** 10.1038/s41598-019-51155-3

**Published:** 2019-10-10

**Authors:** Stéphane Busca, Julia Salleron, Romain Boidot, Jean-Louis Merlin, Alexandre Harlé

**Affiliations:** 1 Service de Biopathologie, Institut de Cancérologie de Lorraine, F-54519 Vandœuvre-lès-Nancy, France; 2 Data Biostatistics Unit, Institut de Cancérologie de Lorraine, F-54519 Vandœuvre-lès-Nancy, France; 3 Department of Tumor Biology and Pathology, Georges-Francois Leclerc Cancer Center – UNICANCER, Dijon, France; 4 Institut National de la Santé et de la Recherche Médicale (Inserm), LNC UMR1231 Dijon, France; 5 Université de Lorraine CNRS UMR 7039 CRAN, F-54519 Vandœuvre-lès-Nancy, France

**Keywords:** Non-small-cell lung cancer, Diagnostic markers

## Abstract

Diagnosis of lung cancer can sometimes be challenging and is of major interest since effective molecular-guided therapies are available. Compounds of tobacco smoke may generate a specific substitutional signature in lung, which is the most exposed organ. To predict whether a tumor is of lung origin or not, we developed and validated the EASILUNG (Exome And SIgnature LUNG) test based on the relative frequencies of somatic substitutions on coding non-transcribed DNA strands from whole-exome sequenced tumors. Data from 7,796 frozen tumor samples (prior to any treatment) from 32 TCGA solid cancer groups were used for its development. External validation was carried out on a local dataset of 196 consecutive routine exome results. Eight out of the 12 classes of substitutions were required to compute the EASILUNG signature that demonstrated good calibration and good discriminative power with a sensitivity of 83% and a specificity of 72% after recalibration on the external validation dataset. This innovative test may be helpful in medical decision-making in patients with unknown primary tumors potentially of lung origin and in the diagnosis of lung cancer in smokers.

## Introduction

Current cancer therapies are generally based on anatomical site. Identifying a tumor’s origin is therefore crucial for treatment selection. Lung cancer is the most common major cancer diagnosed worldwide^1^ and is mostly represented by two subtypes, small cell lung carcinoma and non-small cell lung carcinoma (NSCLC), accounting for 15% and 85% of all lung cancer, respectively^2^. Squamous cell carcinoma (SCC) represents 25 to 30% of lung cancers and adenocarcinoma is the most common histological subtype of lung cancer, representing 40% of all lung cancers. A cancer diagnosed at a metastatic stage with no identifiable primary tumor is termed a cancer of unknown primary origin (CUP). A diagnosis of CUP is evocated when the clinical picture of metastatic disease is associated with one or more tissue biopsy results that are inconsistent with a determined primary tumor. Two to four percent of newly diagnosed cases of carcinoma are CUP^3^; histological subtypes of these CUP are adenocarcinomas (40–60%), undifferentiated carcinomas (15–30%), and SCCs (15–20%)^4^. Patients with CUP syndrome have a median survival of 8–11 months and only 25% survive at 1 year^5^.

The Human Genome Variation Society (HGVS) nomenclature defines 12 different somatic substitution classes (C > A, G > T, C > G, G > C, C > T, G > A, A > C, T > G, A > G, T > C, A > T, and T > A) that can be read on the coding non-transcribed DNA strand. Some childhood cancers, like acute myeloid leukemia (AML), are reported to carry very few somatic substitutions, whereas cancers such as lung carcinomas and malignant melanoma, which are related to chronic exposure to mutagens such as tobacco or ultraviolet light, present with a high number of somatic substitutions^6^. The prevalence of somatic substitutions, quantified in substitutions per megabase (sub/Mb), can be calculated by dividing the number of somatic substitutions by the size of the DNA sequence targeted region in Mb; this is also known as the tumor mutational burden. A mutational signature is a characteristic combination of mutation types arising from specific mutagenesis processes, such as DNA replication infidelity, exposure to exogenous and endogenous genotoxins, defective DNA repair pathways, and DNA enzymatic editing^7^. Tissues directly exposed to tobacco smoke (*e.g*., lung), as well as some tissues not directly exposed (*e.g*., bladder), show elevated levels of DNA adducts in smokers and thus evidence of exposure to carcinogenic components of tobacco smoke^8,9^.Benzo[a]pyrene (BaP) present in tobacco smoke is the most extensively studied compound. After two successive oxidation reactions mediated by cytochrome P450 enzymes (largely CYP1A1), BaP is converted to benzo[a]pyrenediolepoxide (BPDE). BPDE is considered an ultimate carcinogen because, unlike its BaP precursor, it has the ability to directly attack and form covalent adducts with DNA bases, which may then generate G > T/C > A somatic substitutions. Importantly, exposure of a cell to polycyclic aromatic hydrocarbons like BaP and other compounds of tobacco smoke increases synthesis of CYP1A1, thereby accelerating these reactions in cells^10,11^, and may generate a specific substitutional signature in lung, which is the most exposed organ.

The aim of our study was to develop and validate an innovative genomics-based test, the EASILUNG (Exome And SIgnature LUNG) test, able to predict whether or not a tumor is of lung origin using only the prevalence and the relative frequencies of somatic substitutions from whole-exome sequencing (WES) results.

## Methods

### Development dataset

We used the full publicly available TCGA data where somatic mutations are provided as simple somatic mutations (SSM) from WES results. SSM consists of somatic substitutions, deletions of ≤200 bp, insertions of ≤200 bp, and multiple base substitutions (≥2 bp and ≤200 bp). Mutation annotation format (MAF) files containing open access SSM data of the cancer genomes used in this study were obtained from all 33 TCGA groups (February 1, 2016). SSM data from all genome sequencing center (GSC) platforms available were downloaded, corresponding to 130 MAF files containing somatic mutation data from 28,437 WES results from 9,522 distinct tumors sequenced by one to seven GSCs. All TCGA data were generated from frozen tumor samples prior to any treatment. The AML group (2 MAF files containing 394 WES results from 197 distinct samples), WES results with any substitutions (35 WES results), and MAF files that did not include the size of the targeted region of the exome (76 MAF files containing 16,514 WES results) were excluded from the development dataset; these conditions were not mutually exclusive. All WES results from all TCGA solid cancer groups (32/33 TCGA groups) from all GSC platforms that included the size of the targeted region of the exome in MAF files or in previously related TCGA publications were evaluated for the development dataset^12–29^, corresponding to 54 of the 130 MAF files containing somatic mutations in 11,720 WES results from 7,796 of the 9,342 distinct tumors. Each TCGA tumor had a unique barcode; if several GSCs had sequenced the same tumor and there were multiple WES results for the same tumor, a randomization was carried out to select only one WES result for each tumor. In total, the development dataset included 7,796 WES results from 7,796 distinct tumor samples (745 lung cancer and 7,051 non-lung cancer). All details are provided in Supplementary Methods.

### Substitution classes and prevalence of somatic substitutions

Somatic substitutions were analyzed on the coding non-transcribed strand and their absolute frequencies were classified into the 12 classes according to the HGVS nomenclature (C > A, G > T, C > G, G > C, C > T, G > A, A > C, T > G, A > G, T > C, A > T and T > A). Targeted region size (in Mb) and absolute frequencies of the 12 somatic substitution classes read on the coding non-transcribed strand were collected. Insertion, deletion, and multiple base substitutions were not considered. The total number of somatic substitutions was obtained by adding absolute frequencies of the 12 somatic substitution classes. Prevalence of somatic substitutions for each tumor was quantified in sub/Mb and was calculated by dividing the total number of somatic substitutions by the size in Mb of the exome targeted region, which varied between 36 to 64 Mb across TCGA GSCs and groups of solid cancers (Supplementary Methods).

### Relative frequencies of substitution classes

Relative frequencies of somatic substitutions for each class (%CA, %GT, %CG, %GC, %CT, %GA, %AC, %TG, %AG, %TC, %AT, and %TA) were determined as the absolute frequency of each somatic substitution class (respectively C > A, G > T, C > G, G > C, C > T, G > A, A > C, T > G, A > G, T > C, A > T, and T > A) divided by the total number of somatic substitutions.

### External validation dataset

A total of 196 consecutive tumor samples sequenced between January 2015 and August 2017 available from the Molecular Tumor Board of Centre Georges-François Leclerc (CGFL, Dijon, France) was used for the external validation dataset. Informed consent was obtained for each patient and the study was approved by the Centre Georges-François Leclerc ethical board. All methods were performed in accordance with the relevant guidelines and regulations.

All tumors had WES results and matched histopathological characteristics. All tumors and paired germline DNA were sequenced in 2 x 150 cycles on NextSeq. 500 using the Agilent SureSelect XT Human All exon v5 kit (targeted region = 50 Mb). All samples were FFPE or snap-frozen human tumors (primary or metastasis) from different histological classes and localizations. Initial site and diagnoses were determined using histopathological information. For all samples, normal DNA from the same individuals had also been sequenced to establish the somatic origin of variants and exclude germline single nucleotide polymorphisms. For each tumor, a file containing two columns with the allele of reference and mutated allele for each single-base substitution read in the coding non-transcribed strand was sent to our institution (Institut de Cancérologie de Lorraine, France), blinded of all other parameters.

### Identification of the predictive model and establishing the EASILUNG signature

Patients with a somatic substitution prevalence of <1 sub/Mb were classified as having non-lung cancer. For patients with a somatic substitution prevalence of ≥1 sub/Mb, the EASILUNG signature was computed using the 12 substitution classes. They were described by the median and the interquartile range, since the distributions of these parameters were not normal according to the Kolmogorov-Smirnov test. The log-linearity assumption of the logistic model was checked. This was carried out by categorizing each classes of substitution into 10 groups (corresponding to deciles) and by looking at the plot of the logit of observed percentages of lung cancer in each class. When this assumption was not verified, the continuous parameter was transformed into binary variables. This was performed by using the AUC. The threshold maximizing the Youden index was chosen.

A multivariable logistic regression was performed with the 12 substitution classes as independent parameters and the lung cancer as the dependent parameter (full model). The simplification of this full model to avoid overfitting was done using a multivariable logistic regression with backward selection at the level p = 0.2. The stability of the selected model was investigated using the bootstrap resampling method^30^. A multivariable logistic regression was performed using the substitution classes selected by the bootstrap resampling method (final model). Results of the final model were expressed as Odds ratio and 95% confidence interval. Odds ratio correspond to the exponential function of the regression coefficient (eβ). These regression coefficients (β) were multiplied by 100 and rounded to the nearest integer to obtain the EASILUNG score points. These integers were then added to obtain an overall lung cancer signature for each patient, namely the linear predictor. The model’s discriminative performance was assessed by the AUC, and the calibration by the Hosmer-Lemeshow chi-square test. The threshold of the predicted risk score maximizing the Youden index was chosen and the sensitivity and the specificity with their 95% confidence intervals were computed.

The overall performance of the classification was assessed by taking into account the patients with a somatic substitution prevalence of <1 sub/Mb by classifying them as having non-lung cancer, because reports of lung cancers with a <1 sub/Mb prevalence are rare in smokers^6^. The sensitivity and the specificity with their 95% confidence intervals were computed on the total population. The sensitivity corresponded to the number of cases with a score greater than the pre-specified threshold divided by the number of lung cancers. The specificity corresponded to the number of cases with a somatic substitution prevalence of <1 sub/Mb plus the number of cases with a score less than pre-specified threshold divided by the number of non-lung cancer.

### External validation

The EASILUNG score was computed on the validation set. The discriminative performance of the EASILUNG test on the validation set was assessed by the sensitivity and the specificity using the threshold identified with the development dataset. The calibration of the EASILUNG signature was assessed by computing the logistic regression coefficients (the intercept A and the slope B of the regression) using a logistic regression with the presence of lung cancer as dependent variable and unmodified EASILUNG signature as independent parameter. Perfect predictions correspond to an intercept A of 0 and slope B of 1. If this condition is not satisfied, a recalibration has to be performed by computed “A + B × EASILUNG score” giving a recalibrated EASILUNG score^31^. After the recalibration, the discriminative performance was assessed by the AUC and the calibration by the Hosmer-Lemeshow chi-square test. A new threshold maximizing the Youden index was chosen and the sensitivity and the specificity with their 95% confidence intervals were computed.

## Results

### Score development

The development dataset included 7,796 WES results from solid tumors from The Cancer Genome Atlas (TCGA). Among the 7,796 samples, 745 (9.56%) were tumors where the primary origin was lung, from the lung adenocarcinoma group ([LUAD]) or the lung SCC group ([LUSC]) and 7,051 (90.44%) were from the 30 other non-lung primary location groups (Supplementary Table 1). Among these 7,796 samples, 2,888 samples had a somatic substitution prevalence of <1 sub/Mb, including 43 lung cancer cases (1.5%). We considered one lung tumor with a somatic substitution prevalence lower than 1 sub/Mb to be non-lung cancer (because reports of lung cancers with a <1 sub/Mb prevalence are rare). Table 1 describes the 12 substitution classes for the 4,908 tumors with a somatic substitution prevalence of ≥1 sub/Mb, including 702 (14.3%) lung tumors and 4,206 (85.70%) non-lung tumors. The bivariate analyses demonstrated that all classes of substitutions were predictive of lung cancer (Table 2). A relative frequency of C > A (%CA) greater than 12% or of G > T (%GT) greater than 13% were associated with a greater risk of lung cancer (respectively with an odds ratio and 95%CI of 14.44 [11.95;17.46] and 9.17 [7.66;10.98]) whereas the presence of C > G or G > C were associated with a lower risk of lung cancer (respectively 0.45 [0.38;0.54] and 0.50 [0.42;0.60]). The full multivariate model to predict lung cancer including the 12 substitution classes is reported in Table 2.

**Table 1:** Distribution of the relative frequencies of the 12 substitution classes in the development dataset for the 4,908 tumors with a somatic substitution prevalence of ≥1 sub/Mb.

	All	Other location	Lung cancer	p-value
	(n = 4908)	(n = 4206)	(n = 702)	
%CA >12	13.47% (661)	7.01% (295)	52.14% (366)	<0.0001
%GT >13	15.14% (743)	9.49% (399)	49% (344)	<0.0001
%CG ≥1	82.46% (4047)	84.38% (3549)	70.94% (498)	<0.0001
%GC ≥1	81.32% (3991)	83.05% (3493)	70.94% (498)	<0.0001
%CT, median (IQR)	22 (15; 30)	24 (18; 31)	11 (0; 16)	<0.0001
%GA, median (IQR)	22 (15; 30)	25 (18; 32)	11 (0; 16)	<0.0001
%AC, median (IQR)	1 (0; 2)	1 (0; 3)	1 (0; 1)	<0.0001
%TG, median (IQR)	1 (0; 3)	1 (0; 3)	0 (0; 1)	<0.0001
%AG >5	43.44% (2132)	47.81% (2011)	17.24% (121)	<0.0001
%TC >5	42.97% (2109)	47.0% (1977)	18.8% (132)	<0.0001
%AT >3	26.67% (1309)	24.82% (1044)	37.75% (265)	<0.0001
%TA >3	26.55% (1303)	24.37% (1025)	39.6% (278)	<0.0001

Results presented as percentage and frequency %(n) unless otherwise indicated. IQR: interquartile range; %CA = relative frequency of C > A substitutions (%); %GT = relative frequency of G > T substitutions (%); %CG = relative frequency of C > G substitutions (%); %GC = relative frequency of G > C substitutions (%); %CT = relative frequency of C > T substitutions (%); %GA = relative frequency of G > A substitutions (%); %AC = relative frequency of A > C substitutions (%); %TG = relative frequency of T > G substitutions (%); %AG = relative frequency of A > G substitutions (%); %TC = relative frequency of T > C substitutions (%); %AT = relative frequency of A > T substitutions (%); %TA = relative frequency of T > A substitutions (%).

**Table 2:** Bivariate analysis and full multivariate model with the 12 substitution classes using logistic regression for the 4,908 tumors with a somatic substitution prevalence of ≥1 sub/Mb.

Relative frequencies of substitutions in tumors	Bivariate Analyses		Full Mutivariate Model	
	Odds ratio and 95% CI	p-value	Odds ratio and 95% CI	p-value
%CA >12 vs ≤12	14.44 [11.95; 17.46]	0.001	4.86 [3.59; 6.58]	0.001
%GT >13 vs ≤13	9.17 [7.66; 10.98]	<0.001	1.62 [1.2; 2.19]	0.001
%CG ≥1 vs 0	0.45 [0.38; 0.54]	<0.001	1.52 [0.75; 3.06]	0.24
%GC ≥1 vs 0	0.50 [0.42; 0.60]	<0.001	1.58 [0.78; 3.22]	0.20
%CT	0.84 [0.83; 0.85]	<0.001	0.91 [0.88; 0.93]	<0.001
%GA	0.84 [0.83; 0.85]	<0.001	0.93 [0.91; 0.95]	<0.001
%AC	0.67 [0.63; 0.72]	<0.001	0.82 [0.76; 0.90]	<0.001
%TG	0.65 [0.61; 0.70]	<0.001	0.83 [0.76; 0.90]	<0.001
%AG >5 vs ≤5	0.23 [0.19; 0.28]	<0.001	0.43 [0.33; 0.58]	<0.001
%TC >5 vs ≤5	0.26 [0.21; 0.32]	<0.001	0.44 [0.33; 0.59]	<0.001
%AT >3 vs ≤3	1.84 [1.55; 2.17]	<0.001	0.99 [0.74; 1.33]	0.96
%TA >3 vs ≤3	2.04 [1.72; 2.40]	<0.001	1.15 [0.86; 1.53]	0.36

CI = confidence interval; vs = versus; %CA = relative frequency of C > A substitutions (%); %GT = relative frequency of G > T substitutions (%); %CG = relative frequency of C > G substitutions (%); %GC = relative frequency of G > C substitutions (%); %CT = relative frequency of C > T substitutions (%); %GA = relative frequency of G > A substitutions (%); %AC = relative frequency of A > C substitutions (%); %TG = relative frequency of T > G substitutions (%); %AG = relative frequency of A > G substitutions (%); %TC = relative frequency of T > C substitutions (%); %AT = relative frequency of A > T substitutions (%); %TA = relative frequency of T > A substitutions (%).

**Figure 1:**
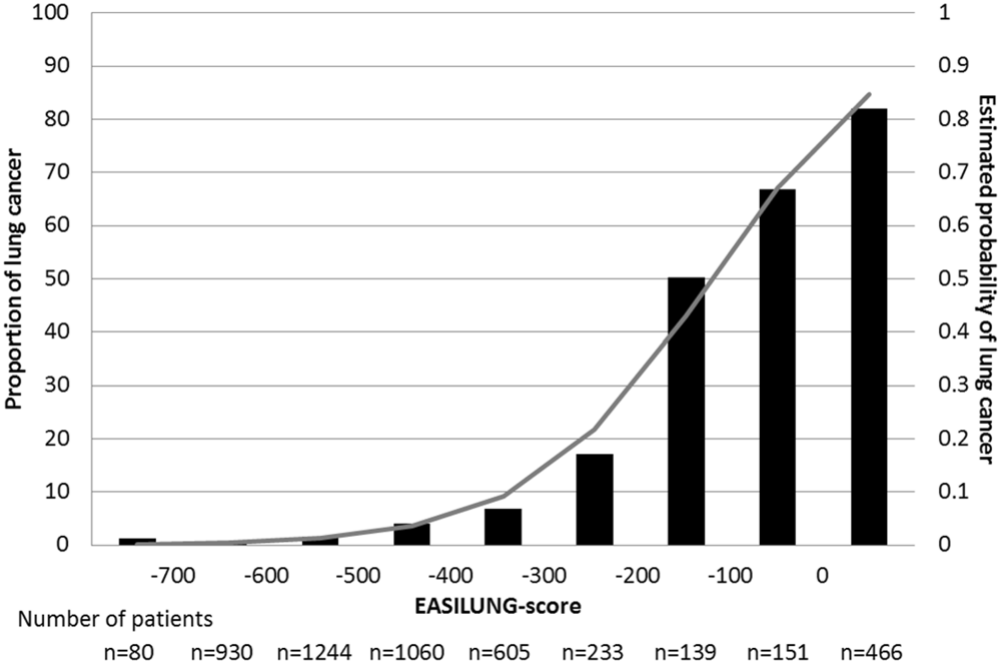
Description of the EASILUNG score in the development dataset. This includes the 4,908 tumors with a somatic substitution prevalence of ≥1 sub/Mb and shows the estimated probability of lung cancer calculated from the EASILUNG score according to the values of the score. Logit (lung cancer) = 1.216 + 0.010 × EASILUNG score. Probability of lung cancer = 1/(1 + exp [−logit(lung cancer)]).

After backward selection with bootstrap validation, eight classes of substitutions were significantly associated with lung cancer and were retained for the EASILUNG test (Table 3). Thus, the final score is defined as Score = +175(if %CA > 12) + 62(if %GT > 13) − 9*(%CT) − 6*(%GA) − 17*(%AC) − 74(if %AG > 5) −73(if %TC > 5). Table 4 details the signature based on these eight classes of substitutions with two examples of how it was calculated. To illustrate the application of the EASILUNG test (Example 1, Table 4), when we considered a tumor with %CA = 27, %GT = 19, %CT = 8, %GA = 12, %AC = 0, %TG = 0, %AG = 3, and %TC = 5, the EASILUNG score was 93 and the estimated predicted probability that the tumor was lung cancer was 0.89, according to Fig. 1.

**Table 3:** Final logistic regression defining the EASILUNG score.

	Odds Ratio^‡^ and 95% CI	β Coefficient^‡^	EASILUNG score points^◊^
%CA >12 vs ≤12	5.78 [4.36; 7.66]	1.7547	175
%GT >13 vs ≤13	1.86 [1.4; 2.47]	0.6209	62
%CT	0.92 [0.9; 0.94]	−0.0863	−9
%GA	0.94 [0.92; 0.96]	−0.0631	−6
%AC	0.84 [0.77; 0.92]	−0.1719	−17
%TG	0.84 [0.78; 0.92]	−0.1704	−17
%AG >5 vs ≤5	0.48 [0.36; 0.63]	−0.7433	−74
%TC >5 vs ≤5	0.48 [0.36; 0.64]	−0.7317	−73

^‡^Odds ratio correspond to the exponential function of the regression coefficient (e^β^).

^◊^β Coefficient*100 rounded to the nearest integer

CI = confidence interval; %CA = relative frequency of C > A substitutions (%); %GT = relative frequency of G > T substitutions (%); %CG = relative frequency of C > G substitutions (%); %GC = relative frequency of G > C substitutions (%); %CT = relative frequency of C > T substitutions (%); %GA = relative frequency of G > A substitutions (%); %AC = relative frequency of A > C substitutions (%); %TG = relative frequency of T > G substitutions (%); %AG = relative frequency of A > G substitutions (%); %TC = relative frequency of T > C substitutions (%); %AT = relative frequency of A > T substitutions (%); %TA = relative frequency of T > A substitutions (%).

**Table 4:** Calculating the EASILUNG score with two examples from the TCGA.

Substitution classes	%CA^∆^	%GT^◊^	%CT	%GA	%AC	%TG	%AG^*^	%TC^‡^	
EASILUNG signature =	+175	+62	−9*(%CT)	−6*(%GA)	−17*(%AC)	−17*(%TG)	−74	−73	
Example 1^(a)^									
*Relative frequency*	*27*	*19*	*8*	*12*	*0*	*0*	*3*	*5*	
EASILUNG score =	+175	+62	−72	−72	+0	+0	+0	+0	=93
Example 2^(b)^									
*Relative frequency*	*2*	*2*	*27*	*37*	*4*	*1*	*6*	*5*	
EASILUNG score =	+0	+0	−243	−222	−68	−17	−74	+0	=−624

%CA = relative frequency of C > A substitutions (%); %GT = relative frequency of G > T substitutions (%); %CT = relative frequency of C > T substitutions (%); %GA = relative frequency of G > A substitutions (%); %AC = relative frequency of A > C substitutions (%); %TG = relative frequency of T > G substitutions (%); %AG = relative frequency of A > G substitutions (%); %TC = relative frequency of T > C substitutions (%).

^∆^For samples with %CA > 12.

^◊^For samples with %GT > 13.

*For samples with %AG > 5.

^‡^For samples with %TC > 5.

^(a)^Example 1/Tumor sample barcode = TCGA-75–7027–01A-11D-1945–08 (lung cancer) with 27% C > A substitutions (+175 since > 12), 19% G > T substitutions(+62 since > 13), 8% C > T substitutions (−9*8), 12% G > A substitutions (−6*12), 0% A > C substitutions (−17*0), 0% T > G substitutions (−17*0), 3% A > G substitutions (0 since ≤5), 5% T > C substitutions (0 since ≤5).

^(b)^Example 2/Tumor sample barcode = TCGA-75–7027–01A-11D-1945–08 (non-lung cancer) with 2% C > A substitutions (+0 since ≤12), 2% G > T substitutions(+0 since ≤ 13), 27% C > T substitutions (−9*27), 37% G > A substitutions (−6*37), 4% A > C substitutions (−17*4), 1% T > G substitutions (−17*1), 6% A > G substitutions (−74 since>5), 5% T > C substitutions (0 since ≤5).

### EASILUNG test performance on an TCGA development dataset

The median EASILUNG score was −485 (IQR from −582 to −353). The score was significantly higher for lung cancer: 0 (from −139 to 2) vs −511 (from −597 to −411) for non-lung cancer (p < 0.0001). Area under the ROC curve (AUC) of the final model was 0.931 [95%CI 0.920; 0.942]. Descriptive characteristics of the EASILUNG score on the development dataset and the probability of lung cancer are provided in Fig. 1. The calibration assessed by the Hosmer- Lemeshow chi-square test was equal to 10.89 (p = 0.2079). The best EASILUNG score threshold to separate lung and non-lung cancer was set at −303: a score greater than −303 favored lung cancer. The associated sensitivity and specificity were respectively 85.7% [83.2%; 88.3%] and 89.6% [88.7%; 90.6%].

Among the 7,796 samples, when we considered the 2,888 samples with a somatic substitution prevalence of <1 sub/Mb to be non-lung cancer, the respective overall sensitivity and overall specificity of the EASILUNG-test were 80.0% [77.1%; 82.8%] (596/745) and 94.3% [93.7%; 94.8%] (6,646/7,051).

Performance of the EASILUNG test thresholded at −303 on the development dataset are presented for lung adenocarcinoma and lung SCC according to patients’ smoking status, and for all other solid cancer groups and subgroups in Supplementary Table 2.

### Test performance on external validation dataset

**Figure 2:**
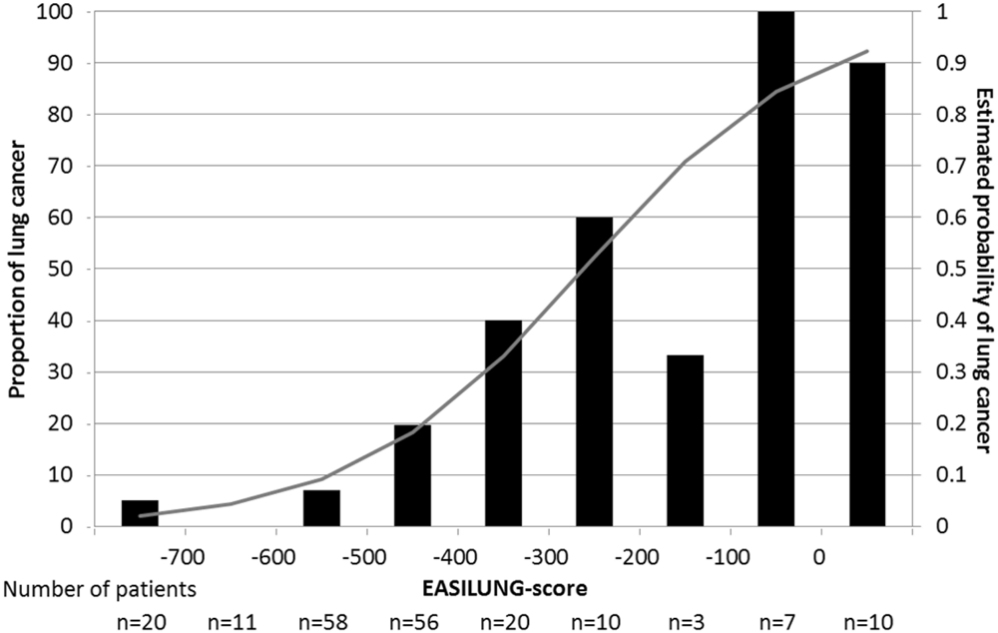
Description of the EASILUNG score in the validation dataset. This includes the 195 tumors with a somatic substitution prevalence of ≥1 sub/Mb and shows the estimated probability of lung cancer calculated from the EASILUNG signature according to the values of the score. Logit (lung cancer) = 2.094 + 0.008 × EASILUNG score. Probability of lung cancer = 1/(1 + exp [−logit(lung cancer)]).

Characteristics of 196 samples and distribution of the relative frequencies of the 12 substitution classes are presented in Supplementary Tables 3 and 4. Among 196 available tumors with WES data, we considered one lung tumor with a somatic substitution prevalence lower than 1 sub/Mb to be non-lung cancer. The EASILUNG score was computed on the remaining 195 samples. Before recalibration by using the threshold of −303, the associated sensitivity and specificity were respectively 48.9% [34.6%; 63.2%] and 95.3% [91.8%; 98.7%]. Descriptive characteristics of the EASILUNG score on the validation dataset and the probability of lung cancer are provided in Fig. 2. The new calibration of EASILUNG score was 2.094 + 0.008 × EASILUNG score. After calibration on the validation dataset, the AUC of the EASILUNG score was 0.849 [0.781; 0.917]. The calibration assessed by the Hosmer-Lemeshow chi-square test was equal to 8.63 (p = 0.3745) with close agreement between predicted and observed risk of lung cancer, with no apparent over- or underprediction (Supplementary Fig. 1). When using the new threshold of −467 calculated after the calibration, the associated overall sensitivity and specificity were respectively 83.0% [72.2%; 93.7%] and 72.3% [65.1%; 79.5%]. Se and Sp were respectively 88.2%[77.4%; 99.1%] and 70.9%[62.9%; 78.8%] for the 161 FFPE samples and 64.3%[39.2%; 89.4%] and 80.9%[64.2%; 97.7%] for the 35 fresh frozen samples without any difference on sensitivities (p = 0.095) nor specificities (p = 0.339). The associated performance of the EASILUNG test on the validation dataset is detailed in Supplementary Table 5.

## Discussion

Lung carcinogenesis by tobacco smoke induce a characteristic pattern of substitutions, our goal was to focus on somatic substitutions to exhibit how these parameters may be helpful to discriminate lung cancer from other cancers, especially in patient exposed to tobacco smoke. EASILUNG test use the relative frequency of somatic substitutions from WES results as a diagnostic tool. Using TCGA data, Alexandrov *et al*.^6^ published a comprehensive signature classification based on the different patterns of genomic substitutions in cancer. In their study, they describe signatures with 96 parameters, corresponding to the type of substitution in their trinucleotide context, and in particular a tobacco-induced signature. These 96-parameter signatures are descriptive and are not actually used clinically in diagnosis, because each is present in several classes of cancer, *e.g*. the signature proposed for tobacco is present in the same way in lung cancers, head and neck SCCs, and in liver hepatocellular carcinoma^6^, without specified thresholds and therefore without assessment performed for the sensitivity and specificity of these signatures. Based on only 8 variables, here we propose a score that can be easily applied to whole-exome results in cases where the somatic substitution prevalence is ≥1 sub/Mb. The 8 substitutions retained for the score are the signature of tobacco related lung cancer.

We stated the threshold of 1 sub/Mb as a hypothesis for this work, as we assumed that tobacco related cancers were more likely to carry at least one somatic mutation per megabase, as described in several publications. This threshold allows an easy selection of potential candidate tumors for the application of the score. The interest of this threshold is to have a minimal number of substitutions allowing a statistical analysis using 12 variables (at least 40 substitutions considering exome targeted region >40 Mb). Furthemore, tobacco-induced lung cancer with <1 sub/Mb is uncommon, in opposite to lung cancer in non-smokers. Finally, this threshold minimizes the artifacts of non-smokers lung tumors data in a score which is related to tobacco smoke. By a posteriori assessment, we noticed that this threshold is the value that allows to reach the best NPV (98.5%). Moreover, the threshold of 1 sub/Mb on an exome sequence makes possible to have an acceptable number of substitutions - at least forty if the exome targeted region is >40 Mb - making the analysis of the distribution of 12 variables statistically significant.

We decided to use the 12 somatic substitution classes in order to consider transcriptional strand biases which is not possible with the regular 6-subtypes approach and also avoid to use the 96-subtypes model to develop a tool easy to use in everyday practice. Strand bias of tobacco-induced mutagenesis could be indicative of specific repair processes that are active in the cell; the mutational signature associated with lung cancer exhibited predominantly G > T/C > A substitutions with a transcriptional strand bias (more G > T transversions than complementary C > A somatic substitutions in the coding non-transcribed strand), suggesting the formation of bulky adducts on guanine and previously described by *Alexandrov et al*.^6^. The efficiency of DNA damage and DNA maintenance processes differ between the transcribed and non-transcribed gene strands. A well-known cause of this phenomenon is transcription-coupled nucleotide excision repair (NER) that operates predominantly on the transcribed strand of genes and is initiated by RNA polymerase II when it encounters bulky DNA helix-distorting lesions^32^. The high rate of G > T/C > A somatic substitutions in lung cancer is probably an imprint of the bulky DNA adducts generated by polycyclic hydrocarbons found in tobacco smoke and their removal by transcription-coupled NER^33^. Furthermore, in our study high C > A relative frequency (%CA > 12%) was the most discriminative parameter, with the highest odds ratio, followed by high G > T relative frequency (%GT > 13%). The higher prevalence of G > T substitutions on the non-transcribed strand compared to complementary C > A substitutions in lung cancer reflects this strand bias and confirms that bulky adduct damage to guanine may be the cause of the observed mutations.

The AML cancer group was not included simply because there is no possible diagnostic confusion between AML and solid cancers in routine clinical practice. Furthermore, AML genomes have fewer mutations than most other adult cancers, with an average of only 13 mutations found^34^. Because of the low density of somatic substitutions in AML (<1 sub/Mb), to reject AML the specificity of our test would have be equal to 100%. Inclusion of AML would have caused inflation of the specificity of our test without any clinical relevance.

No somatic substitution was described in 35 tumors (1 lung SCC and 34 non-lung tumors), accounting for approximately one tumor out of 1000 in TCGA. We assumed that these tumors were simply carrying too few mutations or may have somatic mutations with low allele frequencies that may be excluded from the variants list^35,36^. We thus decided to exclude these 35 tumors from our validation dataset, even though our calculated specificity would have been artificially better, since all of these 35 tumors would have been considered as negative by the EASILUNG test (<1 sub/Mb).

We chose to make distinct TCGA histological subgroups, smoking status subgroups and immunophenotypical subgroups (see Supplementary Table 2) relevant in the differential diagnosis, because some TCGA groups are very heterogeneous. Heterogeneity of the results in lung cancer subgroups according to smoker status (Supplementary Table 2) suggest a high potential for our test in smokers: in lung adenocarcinoma group, positivity of the test is higher than 90% in current reformed smoker for ≤15 years and current smoker (includes daily smokers and non-daily smokers or occasional smokers) subgroups and decrease to 38% in lifelong Non-smoker subgroup (less than 100 cigarettes smoked in lifetime). We also specified anatomical subdivision in the head and neck SCC group because epidemiological and etiological heterogeneities exist in these subgroups although the histological type is still SCC. The larynx is the anatomic region closer to the trachea than other anatomic regions of head and neck SCC. It is therefore clinically relevant to evaluate the results of our test in these different anatomical subgroups. Positive EASILUNG tests in SCCs localized in the larynx (30/61; 49%), which suggests this location is more closely similar to lung SCCs than other head and neck SCC locations: oral cavity (6/153; 4%), oropharynx (2/27; 7%), and hypopharynx (0/3; 0%). The gradient in test positivity through the airways in the head and neck SCC group may reflect the existence of an underlying, parallel gradient in the extent of exposure of cells of the respiratory tract to BaP and other tobacco smoke carcinogens. These results can be linked with a previous study^33^ about a gradient in the prevalence of TP53 G > T transversions in cancers of smokers, from low in the oral cavity, to intermediate in the larynx, and high in various histological types of lung cancers. The EASILUNG score is not limited in discriminating smoking lung cancer from non-smoking cancers. This score is also able to discriminate smoking lung cancer from other localizations independently of the tobacco status. For example (Supplementary Table 2), patients with lung cancer that are current reformed smoker for ≤15 years or current smoker (includes daily smokers and non-daily smokers or occasional smokers) have a positive score in 93% and 91% respectively and patients with head and neck cancer or bladder which are also often smoking-related have a positive score in only 14% and 6% respectively.

Some RNA-based molecular tests studying mRNA expression exist to help identify cases not resolved by immunohistochemistry and classified as CUP. More recently, an alternative strategy was to propose a treatment targeting the supposed primary tumor according to the molecular signature, based on gene expression analyzed by a 92-gene RT-PCR assay^37^. In this study, the sensitivity was 63% for lung adenocarcinoma from frozen samples and 0% for formalin fixed paraffin-embedded (FFPE) samples. One of the hypotheses explaining the limitations of diagnostic methods studying mRNA expression is that a genetic signature predisposing to metastasis coexists in primary tumors with the tissue-specific genetic signature. This “metastatic” genetic program that transcends distinctions between the original tissues has been described and could lead to errors in the molecular classification of various tumors at primary sites such as breast or bladder^38^. The selection of genes relies heavily on the number and type of primary solid tumors studied. In most published series, the “training set” included fewer than 1000 tumor specimens (typically 100–400). In addition, gene expression profiles are heterogeneous in pulmonary adenocarcinomas^39^. By studying DNA, our approach is not impacted by these limitations; this explains our good results and suggests the superiority of our approach in lung cancers, particularly in adenocarcinomas. By analyzing lung adenocarcinomas and lung SCC, we cover the two most prevalent lung cancer histologies and our results suggest that the EASILUNG test remained acceptably sensitive and strongly specific on FFPE samples. The mutational artefact potentially generated by formalin fixation^40^ was not a limitation for our test, as demonstrated by our local sample results. Indeed, formalin crosslinking with cytosine nucleotides on either strand can result in incorrect incorporation of adenine in place of guanosine, causing an artificial C > T/G > A mutation^41,42^ that could impact negatively the score (Table 2).

A known major issue of external validation studies is that they are often based on small and local datasets. This means that there is often heterogeneity in the performance of prediction models and that multiple external validation studies are needed to fully appreciate the generalizability of a model. Our external validation shows that the EASILUNG test will be suitable for other datasets in different platforms without modifying the signature but after a recalibration in each platform before a routine use: this recalibration per platform is required to define the specific threshold to be used. Our diagnosis approach could allow the detection of lung cancer using circulating tumor DNA (ctDNA) detection. ctDNA is a promising biomarker for noninvasive assessment of cancer. In a previous study, ctDNA have been detected in 100% of patients with stage II– to IV NSCLC and in 50% of patients with stage I, with 96% specificity for mutant allele fractions down to ~0.02%^43^. Exome-wide analysis of circulating tumor DNA could complement current invasive biopsy approaches to identify single somatic substitutions spectra and density associated with lung cancer in a future blood-based screening strategy.

In conclusion, we developed an innovative genomics-based tool which may be helpful in diagnosis of lung cancer and in medical decision-making for patients with a history of tobacco smoking with unknown primary tumors potentially of lung origin. The external validation of our test confirmed its great potential. Multiple external validation studies and prospective validation studies on exome results are needed to fully realize the generalizability of our model. Clinical trials are required to evaluate its benefits to patients.

## Data Availability

The local validation sample datasets analyzed during the current study are not fully publicly available: due to reasonable privacy and security concerns, the data are not easily redistributable to researchers other than those engaged in Institutional Review Board-approved research collaborations with the named medical center.
